# Working to improve survival and health for babies born very preterm: the WISH project protocol

**DOI:** 10.1186/1471-2393-13-239

**Published:** 2013-12-19

**Authors:** Caroline A Crowther, Philippa F Middleton, Emily Bain, Pat Ashwood, Tanya Bubner, Vicki Flenady, Jonathan Morris, Sarah McIntyre

**Affiliations:** 1 Australian Research Centre for Health of Women and Babies, Robinson Institute, School of Paediatrics and Reproductive Health, The University of Adelaide, Adelaide, Australia; 2 Liggins Institute, The University of Auckland, Auckland, New Zealand; 3 Centre for Translating Research into Practice, Mater Medical Research Institute, Brisbane, Australia; 4 Discipline of Obstetrics, Gynaecology and Neonatology, The University of Sydney, Sydney, Australia; 5 Cerebral Palsy Institute, The University of Notre Dame Australia, Sydney, Australia

**Keywords:** Evidence-based practice, Implementation, Magnesium sulphate, Neuroprotection, Cerebral palsy, Preterm birth

## Abstract

**Background:**

Babies born very preterm (before 30 weeks gestation) are at high risk of dying in their first weeks of life, and those who survive are at risk of developing cerebral palsy in childhood. Recent high-quality evidence has shown that giving women magnesium sulphate immediately prior to very early birth can significantly increase the chances of their babies surviving free of cerebral palsy. In 2010 Australian and New Zealand clinical practice guidelines recommended this therapy. The WISH (Working to Improve Survival and Health for babies born very preterm) Project aims to bi-nationally improve and monitor the use of this therapy to reduce the risk of very preterm babies dying or having cerebral palsy.

**Methods/Design:**

The WISH Project is a prospective cohort study. The 25 Australian and New Zealand tertiary level maternity hospitals will be provided with a package of active implementation strategies to guide the introduction and local adaptation of guideline recommendations. Surveys will be conducted at individual hospitals to evaluate outcomes related to local implementation progress and the use and value of the WISH implementation strategies. For the hospitals participating in the ‘WISH audit of uptake and health outcomes data collection’, the primary health outcomes (assessed through case note review, and 24 month corrected age questionnaires) will be: the proportion of eligible women receiving antenatal magnesium sulphate; and rates of death prior to primary hospital discharge and cerebral palsy at two years corrected age in infants born to eligible mothers. For hospitals wishing to assess factors influencing translation locally, barriers and facilitators will be measured through interviews with health care professionals, to further guide implementation strategies. Study outcomes for the early phase of the project (Year 1) will be compared with the later intervention phase (Years 2 and 3).

**Discussion:**

The WISH Project will offer insight into the effectiveness of a multifaceted implementation strategy to improve the uptake of a novel neuroprotective therapy in obstetric clinical practice. The successful implementation of antenatal magnesium sulphate for fetal neuroprotection in Australia and New Zealand could lead to over 90 fewer very preterm babies dying or suffering the long-term consequences of cerebral palsy each year.

## Background

### Preterm birth and cerebral palsy: the burden of disease

Babies born very early are at high risk of dying in their first few weeks of life, or if they survive, they may have damage to their developing brain, manifesting as cerebral palsy, blindness, deafness or cognitive dysfunction [1-3]. Over 600 children in Australia and over 150 in New Zealand are diagnosed with cerebral palsy each year [4]; with approximately 40% of all cases being related to preterm birth [5]. The rate of cerebral palsy among neonatal survivors born at less than 28 weeks gestation is up to 30 times higher compared with infants born at term [6]. It is estimated that the cost to the Australian community of cerebral palsy alone (including financial cost and lost wellbeing) is almost AUD$4 billion per annum [4]. Preventing cerebral palsy and identifying causes have been stated as lead priorities for research by consumers, clinicians and researchers [7].

### Magnesium sulphate and prevention of cerebral palsy: the evidence base

Nearly twenty years ago, a case–control study first described the association between antenatal magnesium prior to preterm birth and the prevention of cerebral palsy [8]. The updated Cochrane review ‘Magnesium sulphate for women at risk of preterm birth for neuroprotection of the fetus’ [9], including five randomised controlled trials, showed for the first time that magnesium sulphate given to women prior to preterm birth can reduce the risk of death or cerebral palsy (risk ratio 0.85, 95% confidence interval 0.74 to 0.98; 4446 infants), and of cerebral palsy alone (risk ratio 0.68, 95% confidence interval 0.54 to 0.87; 6145 infants). The Cochrane review showed that 63 women need to be treated with antenatal magnesium sulphate for neuroprotection for one baby to avoid cerebral palsy. The corresponding number needed to treat to benefit for combined death or cerebral palsy was 42 [9]. This was a very important finding, as few interventions to date have been found to prevent the devastating and lifelong consequences of cerebral palsy.

### Clinical practice guidelines for the use of antenatal magnesium sulphate for neuroprotection of the fetus, infant and child

Researchers at the Australian Research Centre for Health of Women and Babies (ARCH) within the Robinson Institute at the University of Adelaide, led a rigorous guideline development process, culminating in the 2010 National Health and Medical Research Council (NHMRC) endorsed Australian and New Zealand clinical practice guidelines for magnesium sulphate for women at risk of early preterm (less than 30 weeks gestation), imminent birth (planned or definitely expected within 24 hours) [10]. These clinical practice guidelines *‘Antenatal magnesium sulphate prior to preterm birth for neuroprotection of the fetus, infant and child’*[10] provide detailed guidance in the form of nine evidence-based recommendations and six good practice points on how and when to use antenatal magnesium sulphate prior to very preterm birth for fetal neuroprotection (Table 1).

**Table 1:** **Summary of clinical recommendations from the NHMRC endorsed Australian and New Zealand clinical practice guidelines **[10]

**Clinical recommendations**	**Grade^**	**Chapter**
**In women at risk of early preterm imminent birth, use magnesium sulphate for neuroprotection of the fetus, infant and child:**	A	4-7
• When gestational age is less than 30 weeks.	B	8
• When early preterm birth is planned or definitely expected within 24 hours (when birth is planned, commence magnesium sulphate as close to four hours before birth as possible).	A	9
• Intravenously with a 4 gram loading dose (slowly over 20–30 minutes) and 1 gram per hour maintenance dose via intravenous route, with no immediate repeat doses. Continue regimen until birth or for 24 hours, whichever comes first.	C	10
• Regardless of plurality (number of babies in utero).	B	11
• Regardless of the reason women (at less than 30 weeks gestation) are considered to be at risk of preterm birth.	B	12
• Regardless of parity (number of previous births for the woman).	B	13
• Regardless of anticipated mode of birth.	B	14
• Regardless of whether or not antenatal corticosteroids have been given.	B	15

^These grades are based on the NHMRC’s standards for grading recommendations for developers of guidelines, and reflect the degree of trust that can be placed in a particular recommendation based on the strength of the evidence (with A indicating the highest degree of trust).

### Translating research recommendations into clinical practice

Even with up-to-date and relevant guidelines, it was not anticipated that health professionals caring for women at risk of an early preterm birth would immediately begin using antenatal magnesium sulphate for fetal neuroprotection without some form of active implementation strategy (apart from passive dissemination). The slow uptake of antenatal corticosteroids for anticipated preterm birth into clinical practice clearly illustrated that “the transfer of valid and relevant research findings into routine practice is unpredictable and tends to be slow and haphazard” [11]. Consequences of slow adoption into practice can be dire. For example, if the change from the initial 20% to 75% uptake of antenatal corticosteroids had happened more rapidly, over 400 additional babies’ lives would have been saved each year in England and Wales [12].

For antenatal magnesium sulphate, an unparalleled opportunity was presented to close an evidence-practice gap early in the research and implementation cycle, and in doing so, increase the likelihood of babies born very preterm surviving free from cerebral palsy. We propose to do this by initiating and implementing strategies shown to be effective in increasing the uptake of appropriate care [13-20].

Implementation often follows identification of evidence-practice gaps and/or variation in practice. Barriers to the uptake of evidence can operate at many levels (the innovation itself; individual professionals or consumers; and social, organisational, economic and political contexts). There are also many categories of barriers – feasibility, accessibility, knowledge, motivation, resources, organisational culture, financial arrangements are just some of these [21,22]. While it was considered likely that multifaceted practice change interventions would be more effective than single interventions, the optimal combinations are not yet known for many clinical settings – including obstetric clinical practice [13]. The interventions selected for this project were thus chosen on the basis of resulting in likely benefit (increased uptake of the recommended treatment) and ability to integrate the interventions into the setting of caring for women about to give birth very early.

### Summary

• High-quality research evidence has clearly shown that antenatal magnesium sulphate prior to imminent, early preterm birth significantly increases the chances of children surviving free of cerebral palsy;

• Antenatal magnesium has not previously been used routinely for the indication of fetal, infant and child neuroprotection;

• With this ‘translational flashpoint’, implementation interventions are needed to promptly increase uptake into routine care, thereby preventing unnecessary deaths or children surviving with cerebral palsy;

• This translation process is likely to be accelerated with active implementation strategies compared with passive dissemination, and by identifying and addressing barriers to implementation;

• This project will offer active implementation interventions (and assess the effectiveness of these translational interventions), monitor rates of appropriate uptake of antenatal magnesium sulphate prior to early preterm birth, and monitor deaths of infants prior to primary hospital discharge and rates of cerebral palsy in early childhood. In the longer-term, there may be the ability to assess the impact on cerebral palsy in later childhood for this cohort (subject to the availability of future funding).

### Aims

The overall aim of this project is to optimise the care of women at risk of very early preterm birth and so improve the chances of survival and long-term good health for their preterm babies. The project will also provide data from a cohort of mothers and their babies born early that will allow monitoring of the use of a new therapy and implementation strategies for the prevention of cerebral palsy, assess the changes in mortality and morbidity, and provide clinical indicators for care that can be used for quality improvement within participating hospitals.

The specific aims of this project are to improve and monitor uptake of the use of antenatal magnesium sulphate as a neuroprotective therapy immediately prior to imminent (birth planned or definitely expected within 24 hours), early preterm birth (less than 30 weeks gestation) [10], to reduce the risk of very preterm babies dying or having cerebral palsy.

## Methods/Design

A prospective cohort study will be conducted. The project will also have a qualitative component which will assess barriers and enablers to changes in practice.

### Entry criteria

#### *Hospital sites eligible to receive active implementation interventions*

All 25 tertiary level maternity hospitals within Australia and New Zealand with a neonatal intensive care unit will be eligible to receive active implementation interventions (Additional file 1). Clinical practice recommendations are that women at risk of an early birth are transferred to an institution with neonatal intensive care facilities. The tertiary level facilities are therefore the most likely sites where magnesium sulphate for neuroprotection will be administered immediately prior to imminent preterm birth, as per the bi-national clinical practice guideline recommendations [10].

All eligible hospitals will be invited to join the project and to nominate a local clinical implementation leader to be a member of the ‘WISH Study Group’ with the aim, at their institution, of promoting the use of antenatal magnesium sulphate as a fetal neuroprotective therapy, prior to imminent, early preterm birth; with the option of participating in the ‘WISH audit of uptake and health outcomes data collection’, monitoring uptake, mortality and cerebral palsy.

#### *Women eligible at hospital sites participating in the ‘WISH audit of uptake and health outcomes data collection’*

Eligible women at hospitals participating in the ‘WISH audit of uptake and health outcomes data collection’ (Additional file 1) will be those giving birth after 22 completed weeks of gestation and before 30 weeks gestation (excluding women carrying fetuses with known lethal congenital anomalies).

### Implementation interventions

#### *Active dissemination and strategies to implement the Australian and New Zealand antenatal magnesium sulphate clinical practice guidelines at all eligible hospital sites*

A package of active implementation strategies (chosen on the basis of likely benefit) will include the guided introduction and local adaptation of guideline recommendations, in-service education [14,17], academic detailing [18], reminders [14] and audit and feedback [13,14,16]. The local clinicians will be encouraged to adapt the antenatal magnesium sulphate guidelines in line with their local hospital protocols and produce algorithms to communicate work flow and decision points [13].

Each hospital will also be provided with a WISH Project Guideline Action Pack (GAP). The GAP includes a ‘tool kit’ of information developed by the WISH Study Group to aid in implementation [14,20], including the NHMRC endorsed guidelines, educational materials including a PowerPoint for use in in-services, health professional and consumer information (Additional files 2 and 3), posters (Additional file 4) and reminder hospital record inserts (Additional file 5).

Each hospital will be strongly encouraged to form a local implementation group (or identify a local leader) [13,19], with the actual composition to be determined at the local level. The local implementation group will be guided to use the GAP materials to suit local needs. Formal and informal in-service education sessions will be encouraged [17], as well as one-on-one discussions with health professionals caring for women at risk of very preterm birth to address any individual questions and concerns (academic detailing) [18].

We will use audit and feedback, as this has been identified as an effective strategy [13], particularly where baseline adherence to recommended practice is low and where intensive feedback can be given [16]. A regular newsletter will be sent to all hospitals for distribution to relevant local health professionals highlighting overall progress in the project, and giving information on the latest rates of uptake of magnesium sulphate for neuroprotection from those sites participating in the ‘WISH audit of uptake and health outcomes data collection’.

### Project outcomes

#### *Project outcomes for all eligible hospital sites*

Outcomes relating to local implementation progress and the WISH Project strategies will be evaluated in all eligible hospital sites across Australia and New Zealand during the early phase of the intervention, and again in the later intervention phase:

• Local guideline information; including the proportion of eligible hospital sites following a guideline for antenatal magnesium sulphate;

• Evaluation of uptake; including the proportion of eligible hospital sites evaluating uptake of antenatal magnesium sulphate;

• The perceived usefulness of WISH Project active implementation strategies and materials; including the proportion of eligible hospital sites using the WISH GAP materials; and the proportion of hospital that perceive the WISH GAP materials as useful.

#### *Primary health outcomes for hospital sites participating in the ‘WISH audit of uptake and health outcomes data collection’*

• The proportion of women giving birth after 22 completed weeks gestation and before 30 weeks gestation (excluding known lethal congenital anomalies) receiving antenatal magnesium sulphate (judged to be eligible for such treatment according to the bi-national clinical practice guidelines [10]) during three consecutive years;

• Deaths prior to primary hospital discharge of babies born after 22 completed weeks gestation and before 30 weeks gestation during three consecutive years;

• Cerebral palsy rate at early childhood follow-up (two years corrected age) in babies born alive after 22 completed weeks gestation and before 30 weeks gestation in the first year of three consecutive years. The importance of follow-up of this cohort is recognised; further support will be sought to ensure the timely and ongoing collection of the highest quality follow-up data including at preschool and early school age.

#### *Secondary health outcomes for hospital sites participating in the ‘WISH audit of uptake and health outcomes data collection’*

• Serious adverse events related to administration of magnesium sulphate therapy;

• Total dose of magnesium sulphate administered per woman.

#### *Additional outcomes*

For hospital sites wishing to undertake an in-depth assessment of factors influencing uptake locally, barriers and enablers to the use of antenatal magnesium sulphate as perceived by health care professionals will be assessed during the early phase of the intervention, and again in the later intervention phase.

### Data collection and management

#### *For all eligible hospital sites*

The 25 eligible hospital sites across Australia and New Zealand will be surveyed using a web-based system during the early phase of the intervention, and again in the later intervention phase. The invitation to participate will be directed to a local clinical implementation leader, and will be voluntary, with consent implied by completion of the survey.

The surveys will collect information relating to the following content areas: Demographic information (including participating hospital; position in hospital of respondent); Local guideline information (including whether a local guideline for antenatal magnesium sulphate for fetal neuroprotection is being followed; the nature of the guideline; the availability of the guideline; methods used to release the guideline to health professionals; perceived barriers and enablers to guideline implementation); WISH Project strategies and materials (including whether a local implementation group/leader is in place; whether educational sessions have been held; and the use and usefulness of the GAP materials: PowerPoint presentation, health professional and consumer information brochures, posters and reminder hospital record inserts); and Evaluation of uptake (whether a process in place to evaluate uptake; methods used; and approximations). Questions will largely provide Yes, No and Unsure options, with the opportunity to add comments. Views on WISH Project strategies and GAP materials will be elicited by seeking the level of usefulness using a five-point Likert scale (very useful; somewhat useful; undecided; not really useful; not at all useful).

#### *For hospital sites participating in the ‘WISH audit of uptake and health outcomes data collection’*

All eligible women who give birth after 22 completed weeks gestation and before 30 weeks gestation (excluding known lethal congenital anomalies) at the participating hospitals will be given the project information sheet and counselled by a member of the research team about participating in the project. Women will then be given the opportunity to discuss their participation with their families. Informed, written consent will be obtained by the research team if the woman agrees to participate in the project.

For the woman and her baby, consent to enrol in the project will provide consent to access information from the maternal and neonatal clinical records. Mothers of children who survive to primary hospital discharge will be invited to complete the Ages and Stages Questionnaire at 12 and 24 months corrected age, providing information about their child’s health and development [23].

Information will be collected from the maternal case record on maternal demographic characteristics, mode of birth, reason for preterm birth, use of antenatal corticosteroids, tocolysis, eligibility for magnesium sulphate, and whether magnesium sulphate was given. If magnesium sulphate was given, the reason for magnesium sulphate, total dose administered, gestational age given, whether loading, maintenance or repeat doses were used, and timing prior to birth will be recorded together with any adverse effects. For the infant, stillbirth and death of live-born infants prior to primary hospital discharge will be collected.

Information on early child health will also be sought from the child health record up to two years corrected age, in liaison with the family, from any formal paediatric assessment of motor function and psychological assessment.

#### *For hospital sites undertaking an in-depth assessment of barriers and enablers*

Face-to-face interviews will be conducted with randomly selected, consenting health professionals (obstetric and neonatal) during the early phase of the intervention, and again in the later intervention phase. Interviewees will be asked a range of semi-structured questions regarding their awareness and use of the magnesium sulphate clinical practice guidelines, and perceived factors influencing the uptake of this therapy (including barriers and enablers to use, and suggestions to improve uptake locally).

### Data analyses

#### *For all eligible hospital sites*

WISH Project outcomes for the early intervention phase of the project (Year 1) relating to local implementation of a guideline for antenatal magnesium sulphate and WISH Project strategies and materials will be compared with the later intervention phase (Years 2 and 3).

#### For hospital sites participating in the ‘WISH audit of uptake and health outcomes data collection’

Project outcomes for the early phase of the project (Year 1) will be compared with the later intervention phase (Years 2 and 3). The proportion of eligible women prescribed magnesium sulphate will be analysed with Chi^2^ tests (p values of 0.05 regarded as statistically significant) for the earlier period, compared with the later period. Similarly, the rates of death prior to primary hospital discharge for the earlier project period will be compared with the later period. Individual adverse events will be presented as average rates per woman receiving magnesium sulphate. Uptake of magnesium sulphate and rates of death prior to primary hospital discharge after birth will be graphed over time to assess trends; the proportion of infants born during Year 1 with cerebral palsy (identified by age two) will be compared with proportions identified in the literature; with the opportunity, subject to further funding, to compare with proportions born during Years 2 and 3.

#### *For hospital sites undertaking an assessment of barriers and enablers*

Qualitative data from the semi-structured interviews with health professionals involved in the care of women at risk of an early birth will be transcribed and grouped into themes, using one or more of the theoretical frameworks of behaviour change and motivation, that have been used for analysis of barriers and enablers in the translation from ‘knowing to doing’ [24,25]. Themes from the early intervention phase of the project (Year 1) will be compared with the later intervention phase (Years 2 and 3).

### Ethical considerations

Ethics approval for the WISH Project has been granted by the Children’s Youth and Women’s Health Service (CYWHS) Human Research Ethics Committee at the Women’s and Children’s Hospital, Adelaide, Australia (REC2304/8/13). Ethics approval for participation in the ‘WISH audit of uptake and health outcomes data collection’ has been granted by the local institutional review boards for each participating hospital site (see Additional file 1). Hospital sites wishing to undertake an assessment of barriers and enablers will require ethics approval from their local institutional review boards.

## Discussion

There is strong evidence that antenatal magnesium sulphate given to women prior to early preterm (at less than 30 weeks gestation), imminent birth (when birth is planned or definitely expected within 24 hours) significantly increases the chances of their babies surviving free of cerebral palsy [9,10]. It is anticipated that the active implementation strategies used in this project, believed to be effective in increasing the uptake of appropriate care, will lead to a significant increase in the appropriate use of antenatal magnesium sulphate for women prior to early preterm birth. It is estimated that the successful adoption into clinical practice of this valid and relevant research could lead to over 90 fewer very preterm babies dying or suffering the long-term consequences of cerebral palsy in Australia and New Zealand each year [26,27], and should accordingly reduce the burden of this condition on Australian and New Zealand individuals, families and communities.

## Abbreviations

GAP: Guideline action pack; NHMRC: National health and medical research council; WISH: Working to improve survival and health for babies born very preterm.

## Competing interests

CAC was the principal investigator for the Australasian Collaborative Trial of Magnesium Sulphate (ACTOMgSO4). CAC and PFM were authors of the Cochrane review ‘Magnesium sulphate for women at risk of preterm birth for neuroprotection of the fetus.’ CAC, PFM, TB, VF, JM and SM were members of the Guideline Development Panel, for the Australian NHMRC endorsed ‘Antenatal magnesium sulphate prior to preterm birth for neuroprotection of the fetus, infant and child: National clinical practice guidelines.’ The authors declare that they have no other competing interests.

## Authors’ contributions

CAC and PFM wrote the first draft of the WISH Protocol, and prepared the initial draft of this manuscript with EB. CAC and PFM conceived the project, and all others were involved in the development of the design of the project, the protocol development, and have commented on all drafts of this manuscript. All authors read and approved the final manuscript.

## Pre-publication history

The pre-publication history for this paper can be accessed here:

http://www.biomedcentral.com/1471-2393/13/239/prepub

## Supplementary Material

Additional file 1 **All eligible hospital sites and those participating in the ‘WISH audit of uptake and health outcomes data collection’.** List of all hospital sites eligible to receive WISH active implementation strategies, and list of hospital sites with ethics approval for participation in the ‘WISH audit of uptake and health outcomes data collection’. Click here for file

Additional file 2 **3-Fold Information Sheet: *Summary of clinical recommendations and good practice points for health professionals. ***This brochure can be distributed to all health professionals involved in the care of women at risk of very preterm birth to help raise awareness and use of the clinical practice guidelines. Click here for file

Additional file 3 **3-Fold Patient Information Sheet: *Antenatal magnesium sulphate therapy for improving the health of preterm babies: information for women. ***This brochure can be given to women at risk of a very preterm birth. Click here for file

Additional file 4 **Magnesium sulphate implementation poster.** These posters can be put up in perinatal care areas to assist with implementation of the clinical practice guidelines. Click here for file

Additional file 5 **Case note reminder insert for magnesium sulphate.** These case note inserts can be placed in the medical records of women at risk of very preterm birth to remind clinicians to consider their patient’s eligibility for antenatal magnesium sulphate. Click here for file

## References

[B1] Doyle LW for the Victorian Infant Collaborative Study Group Outcome at 5 years of age of children 23 to 27 weeks’ gestation: refining the prognosis Paediatrics 2001 13 1 134 141 10.1542/peds.108.1.134 11433066

[B2] Saigal S Doyle L An overview of mortality and sequelae of preterm birth from infancy to adulthood Lancet 2008 13 9608 261 269 10.1016/S0140-6736(08)60136-1 18207020

[B3] Laws P Sullivan EA Australia’s Mothers and Babies 2007 2009 Sydney: Australian Institute of Health and Welfare (AIHW) Cat No, PER 48

[B4] Access Economics The Economic Impact of Cerebral Palsy in Australia in 2007 2008 Sydney

[B5] Australian Cerebral Palsy Register Group Report of the Australian Cerebral Palsy Register, Birth Years 1993–2006 2013 Sydney 10.1111/dmcn.13026 26762930

[B6] Stanley F Survival and cerebral palsy in low birthweight infants: implications for perinatal care Paediatr and Perinat Epidemiol 1992 13 2 298 310 10.1111/j.1365-3016.1992.tb00769.x 1584730

[B7] McIntyre S Novak I Cusick A Consensus research priorities for cerebral palsy: a Delphi survey of consumers, researchers, and clinicians Dev Med Child Neurol 2010 13 3 270 275 10.1111/j.1469-8749.2009.03358.x 19694780

[B8] Nelson KB Grether JK Can magnesium sulphate reduce the risk of cerebral palsy in very low birthweight infants? Paediatrics 1995 13 2 263 269 7838646

[B9] Doyle LW Crowther CA Middleton P Marret S Rouse D Magnesium sulphate for women at risk of preterm birth for neuroprotection of the fetus Cochrane Database Syst Rev 2009 13 CD004661 19160238 10.1002/14651858.CD004661.pub3

[B10] Antenatal Magnesium Sulphate for Neuroprotection Guideline Development Panel Antenatal magnesium sulphate prior to preterm birth for neuroprotection of the fetus, infant and child: National clinical practice guidelines 2010 Adelaide: The University of Adelaide

[B11] Penney G Foy R Do clinical guidelines enhance safe practice in obstetrics and gynaecology? Best Prac Res Clin Ob Gyn 2007 13 4 657 673 10.1016/j.bpobgyn.2007.01.014 17418642

[B12] Mugford M Corticosteroids in preterm delivery – estimated impact on neonatal outcomes and health service resources Oxford Regional Health Authority, A review of stillbirths and neonatal mortality in the Oxford Region 1993 Oxford: Oxford Regional Health Authority 37 40

[B13] Chaillet N Dube E Dugas M Audibert F Tourigny C Fraser WD Dumont A Evidence-based strategies for implementing guidelines in obstetrics: a systematic review Obstet Gynecol 2006 13 5 1234 1245 10.1097/01.AOG.0000236434.74160.8b 17077251

[B14] Grimshaw J Eccles M Thomas R MacLennan G Ramsay C Fraser C Vale L Toward evidence-based quality improvement J Gen Intern Med 2006 13 2 S14 S20 16637955 10.1111/j.1525-1497.2006.00357.x PMC2557130

[B15] Grimshaw JM Thomas RE MacLennan G Fraser C Ramsay CR Vale L Whitty P Eccles MP Matowe L Shirran L Wensing M Dijkstra R Donaldson C Effectiveness and efficiency of guideline dissemination and implementation strategies Health Technol Assess 2004 13 6 1 72 10.3310/hta8060 14960256

[B16] Ivers N Jamtvedt G Flottorp S Young JM Odgaard-Jensen J French SD O’Brien MA Johansen M Grimshaw J Oxman AD Audit and feedback: effects on professional practice and health care outcomes Cochrane Database Syst Rev 2012 13 CD000259 22696318 10.1002/14651858.CD000259.pub3 PMC11338587

[B17] Forsetlund L Bjørndal A Rashidian A Jamtvedt G O’Brien MA Wolf FM Davis D Odgaard-Jensen J Oxman AD Continuing education meetings and workshops: effects on professional practice and health care outcomes Cochrane Database Syst Rev 2009 13 CD003030 19370580 10.1002/14651858.CD003030.pub2 PMC7138253

[B18] O’Brien MA Rogers S Jamtvedt G Oxman AD Odgaard-Jensen J Kristoffersen DT Forsetlund L Bainbridge D Freemantle N Davis D Haynes RB Harvey E Educational outreach visits: effects on professional practice and health care outcomes Cochrane Database Syst Rev 2007 13 CD000409 17943742 10.1002/14651858.CD000409.pub2 PMC7032679

[B19] Flodgren G Parmelli E Doumit G Gattellari M O’Brien MA Grimshaw J Eccles MP Local opinion leaders: effects on professional practice and health care outcomes Cochrane Database Syst Rev 2011 13 CD000125 21833939 10.1002/14651858.CD000125.pub4 PMC4172331

[B20] Giguère A Légaré F Grimshaw J Turcotte S Fiander M Grudniewicz A Makosso-Kallyth S Wolf FM Farmer AP Gagnon MP Printed educational materials: effects on professional practice and healthcare outcomes Cochrane Database Syst Rev 2012 13 CD004398 23076904 10.1002/14651858.CD004398.pub3 PMC7197046

[B21] Cabana MD Rand CS Powe NR Wu AW Wilson MH Abboud PA Rubin HR Why don’t physicians follow clinical practice guidelines? A framework for improvement JAMA 1999 13 15 1458 1465 10.1001/jama.282.15.1458 10535437

[B22] National Institute of Clinical Studies Identifying barriers to evidence uptake 2006 Melbourne

[B23] Bricker D Squires J Monts L Ages and Stages Questionnaire. A parent completed child monitoring system 1995 USA: Paul H Brookes Publishing Co

[B24] Michie S Van Stralen MM West R The behaviour change wheel: a new method for characterising and designing behaviour change interventions Implement Sci 2011 13 42 10.1186/1748-5908-6-42 21513547 PMC3096582

[B25] Cane J O’Connor D Michie S Validation of the theoretical domains framework for use in behaviour change and implementation research Implement Sci 2012 13 37 10.1186/1748-5908-7-37 22530986 PMC3483008

[B26] Australian Bureau of Statistics 3301.0 Births, Australia*,* 2012 http://www.abs.gov.au/ausstats/abs@.nsf/mf/3301.0

[B27] Statistics New Zealand Births and death*s*: year ended December 2012 http://www.stats.govt.nz/browse_for_stats/population/births/BirthsAndDeaths_HOTPYeDec12.aspx

