# Supplementary material for: Working to improve survival and health for babies born very preterm: the WISH project protocol
Source: BMC Pregnancy Childbirth. 2013 Dec 19;13:239. doi: 10.1186/1471-2393-13-239 (PMC3879421; doi:10.1186/1471-2393-13-239)
Supplement: Additional file 1 — All eligible hospital sites and those participating in the ‘WISH audit of uptake and health outcomes data collection’. List of all hospital sites eligible to receive WISH active implementation strategies, and list of hospital sites with ethics approval for participation in the ‘WISH audit of uptake and health outcomes data collection’. [file 1471-2393-13-239-S1.pdf]

Australian and New Zealand tertiary maternity hospital sites eligible to receive active implementation interventions:

1. Women's and Children's Hospital, South Australia
2. Flinders Medical Centre, South Australia
3. Canberra Hospital, Australian Capital Territory
4. John Hunter Hospital, New South Wales
5. Liverpool Hospital, New South Wales
6. Nepean Hospital, New South Wales
7. Royal Hospital for Women, New South Wales
8. Royal North Shore Hospital, New South Wales
9. Royal Prince Alfred Hospital Women and Babies, New South Wales
10. Westmead Hospital, New South Wales
11. Royal Darwin Hospital, Northern Territory
12. Mater Mothers' Hospital, Queensland
13. Royal Brisbane and Women's Hospital, Queensland
14. The Townsville Hospital, Queensland
15. Royal Hobart Hospital, Tasmania
16. Mercy Hospital for Women, Victoria
17. Monash Medical Centre, Victoria
18. The Royal Women's Hospital, Victoria
19. King Edward Memorial Hospital for Women, Western Australia
20. Auckland City Hospital, New Zealand
21. Christchurch Hospital, New Zealand
22. Dunedin Hospital, New Zealand
23. Middlemore Hospital, New Zealand
24. Waikato Hospital, New Zealand
25. Wellington Women's Hospital, New Zealand

Hospitals sites with approval to participate in the 'WISH audit of uptake and health outcomes data collection':

1. Women's and Children's Hospital, South Australia: approved by Children, Youth and Women's Health Service (CYWHS) Human Research Ethics Committee
2. Royal North Shore Hospital, New South Wales: approved by Northern Sydney Local Health District (NSLHD) Human Research Ethics Committee
3. Mater Mothers' Hospital, Queensland: approved by Mater Health Services (MHS) Human Research Ethics Committee and MHS and Mater Medical Research Institute (MMRI) Human Research Governance Committee
