# Supplementary material for: Working to improve survival and health for babies born very preterm: the WISH project protocol
Source: BMC Pregnancy Childbirth. 2013 Dec 19;13:239. doi: 10.1186/1471-2393-13-239 (PMC3879421; doi:10.1186/1471-2393-13-239)
Supplement: Additional file 2 — 3-Fold Information Sheet: Summary of clinical recommendations and good practice points for health professionals. This brochure can be distributed to all health professionals involved in the care of women at risk of very preterm birth to help raise awareness and use of the clinical practice guidelines. [file 1471-2393-13-239-S2.pdf]

Antenatal magnesium sulphate prior to preterm birth for neuroprotection of the fetus, infant and child

***** This set of clinical recommendations needs to be considered as a whole – recommendations *should not* be applied in isolation**

Recommendation 1
What gestational age and when?

Grading of recommendation: A

In women at risk of early preterm[1] imminent[2] birth, use magnesium sulphate ($MgSO_4$) for neuroprotection of the fetus, infant and child.

[1] when gestational age is < 30 weeks

[2] when early preterm birth is planned or definitely expected within 24 hours. (When birth is planned, commence $MgSO_4$ as close to four hours before birth as possible)

Good Practice Point

Timing

If birth before 30 weeks is planned or expected to occur sooner than four hours (e.g. scheduled caesarean or late presentation to hospital), administer $MgSO_4$ to women at risk of preterm birth, as there is likely still advantage within this time.

Urgent delivery

In situations where urgent delivery is necessary because of actual or imminent maternal or fetal compromise (e.g. severe fetal distress or antepartum haemorrhage), then birth should not be delayed to administer $MgSO_4$.

Recommendation 2
What to give?

Grading of recommendation: C

$MgSO_4$ intravenously with a 4 gram loading dose (slowly over 20-30 minutes) and 1 gram/hour maintenance dose via IV route, with no immediate repeat doses.

Continue regimen until birth or for 24 hours, whichever comes first.

Good Practice Point

Repeat doses

If birth does not occur after giving $MgSO_4$ for neuroprotection of the infant, and preterm birth (< 30 weeks' gestation) again appears imminent (planned or definitely expected with 24 hours), a repeat dose of $MgSO_4$ may be considered at the discretion of the attending health professional.

Recommendations 3-7
Who to give $MgSO_4$ to?

Grading of recommendation: B

Give regardless of:

- plurality (number of babies *in utero*)
- reason woman (at < 30 weeks' gestation) considered at risk of preterm birth
- parity (number of previous births for the woman)
- anticipated mode of birth
- whether or not antenatal corticosteroids have been given

Good Practice Point

Locations of administration of antenatal $MgSO_4$

Locations of administration of antenatal $MgSO_4$ intravenously (IV) to women should be determined by each individual maternity facility.

Monitoring

While administering $MgSO_4$ intravenously, women should be regularly assessed as detailed in individual obstetric unit protocols.

Resuscitation and ventilatory support should be immediately available while administering $MgSO_4$. Should hypotension or respiratory depression occur prompt medical review is recommended. This may include stopping $MgSO_4$.

Loading infusion

A minimum assessment should include checking:

- pulse
- blood pressure
- respiratory rate
- patellar reflexes

before loading dose, 10 minutes *after* loading dose infusion starts and at the *end* of loading dose infusion (20-30 minutes)

The infusion should be stopped if:

- respiratory rate decreases more than 4 breaths/min below baseline
- respiratory rate is < 12 breaths/min
- diastolic blood pressure decreases more than 15 mm Hg below baseline

Maintenance

While the maintenance infusion is running observe for any adverse effects.

The minimum assessments should include checking 4 hourly:

- pulse
- blood pressure
- respiratory rate
- patellar reflexes
- urine output

Stop infusion if:

- respiratory rate < 12 breaths/min
- patellar reflexes are absent
- hypotension occurs (diastolic BP < 15 mm Hg below baseline)
- urine output < 100 mL over 4 hours

Toxicity

Magnesium toxicity is unlikely with the regimens recommended in these guidelines and serum magnesium concentrations do not need to be routinely measured. In women with renal compromise, serum magnesium monitoring is recommended.

Calcium gluconate (1 g (10 mL of 10% solution) slowly via IV route over 10 minutes) can be given if there is clinical concern over respiratory depression.

Potential interactions

There is a potential theoretical interaction between $MgSO_4$ and nifedipine of hypotension and neuromuscular blockade effects, although this is seldom reported in clinical practice.

Regular monitoring of the mother is recommended as detailed in individual obstetric unit protocols. If hypotension occurs, nifedipine and $MgSO_4$ administration should cease and the woman be reviewed by a medical practitioner.

NHMRC grading of recommendations	
A	Body of evidence can be trusted to guide practice
B	Body of evidence can be trusted to guide practice in most situations
C	Body of evidence provides some support for recommendation(s) but care should be taken in its application
D	Body of evidence is weak and recommendation must be applied with caution

For further information

Antenatal Magnesium Sulphate Prior to Preterm Birth for Neuroprotection of the Fetus, Infant and Child National Clinical Practice Guidelines, http://www.nhmrc.gov.au/publications/synopses/cp128syn.htm

Disclaimer: This summary is for general guidance only and is subject to a clinician's expert judgement. The summary should not be relied upon as a substitute for clinical advice. The Antenatal Magnesium Sulphate for Neuroprotection Guideline Development Panel Antenatal Magnesium Sulphate prior to preterm birth for neuroprotection of the fetus, infant and child. First edition. © The Australian Research Centre for Health of Women and Babies, The University of Adelaide 2010.

27/04/11

ANTENATAL MAGNESIUM SULPHATE PRIOR TO PRETERM BIRTH FOR NEUROPROTECTION OF THE FETUS, INFANT AND CHILD

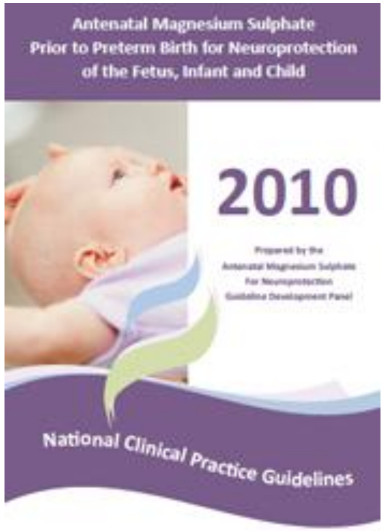

Summary of Clinical Recommendations and Good Practice Points
