# Supplementary material for: Working to improve survival and health for babies born very preterm: the WISH project protocol
Source: BMC Pregnancy Childbirth. 2013 Dec 19;13:239. doi: 10.1186/1471-2393-13-239 (PMC3879421; doi:10.1186/1471-2393-13-239)
Supplement: Additional file 3 — 3-Fold Patient Information Sheet: Antenatal magnesium sulphate therapy for improving the health of preterm babies: information for women. This brochure can be given to women at risk of a very preterm birth. [file 1471-2393-13-239-S3.pdf]

Thank you for taking time to read this leaflet

This gives information about a therapy you might be offered: magnesium sulphate.

Introduction

Every year over 4000 women in Australia give birth very early because of complications with their pregnancy. Babies born before 30 weeks of pregnancy are called very preterm.

What happens if my baby is born early?

Your doctor will discuss the risks from early birth for you and your baby. Very preterm babies have higher risk of complications, including difficulties breathing, coping with infection and with feeding. Most of these problems eventually resolve.

Some very preterm babies have longer term problems that last beyond infancy and childhood, including harm to their brain that may lead to cerebral palsy, blindness or deafness.

What is cerebral palsy?

Cerebral palsy is the term used for a number of different diseases or conditions that involve disorders of movement, posture and motor function. These cause ongoing problems although their exact nature may change over time. Children can be affected to varying degrees and it can take time to work out the extent of their problems. Cerebral palsies can happen any time during the brain's development and are the most common cause of severe motor disability in childhood.

About 600 children are diagnosed with cerebral palsy each year in Australia and just under half of all cases (about 45%) are related to very preterm birth.

Can cerebral palsy be cured?

Unfortunately at present we don't know how to entirely avoid or cure cerebral palsy. The best we can do is to minimise the risk of it happening.

What can be done to prevent cerebral palsy?

Women at risk of a very preterm birth can be given magnesium sulphate therapy to decrease the risk that their baby will develop cerebral palsy.

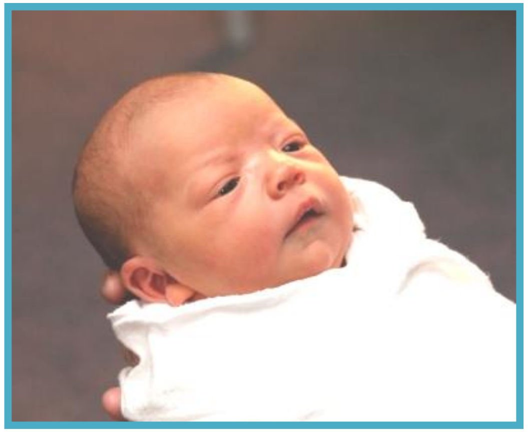

Are there any risks from magnesium sulphate therapy?

Women given magnesium sulphate may get some effects during the therapy, such as flushing, nausea or vomiting, or headaches. Other side effects are less common but will be monitored, such as low blood pressure or problems with breathing.

There is no increased risk to a very preterm baby from magnesium sulphate therapy.

Will I be offered magnesium sulphate therapy?

Your doctor may speak to you about giving you magnesium sulphate if it looks as though you are likely to give birth within 24 hours and it is still early in your pregnancy (before 30 weeks).

You can be given magnesium sulphate therapy:

- Even if you are pregnant with more than one baby
- Whatever the reason for your risk of preterm birth
- However many times you've been pregnant before
- However you expect to give birth (vaginally or by caesarean delivery)
- Whether you've had corticosteroids to help develop your baby's lungs.

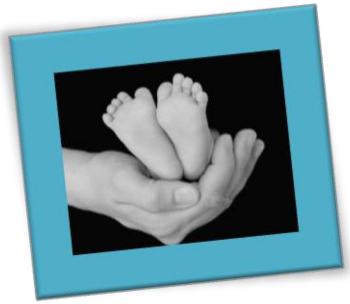

How will I get magnesium sulphate therapy?

Magnesium sulphate is given through a drip (intravenously) over a period of 24 hours so you will need to be in hospital where you can be monitored.

What if birth is urgent?

Your doctor won't delay the birth to give magnesium sulphate.

What if birth doesn't occur within 24 hours?

Your doctor may consider giving you another dose of magnesium sulphate if the birth is delayed.

What if I choose not to receive the therapy?

If you choose not to receive magnesium sulphate therapy, it will not affect your or your baby's care in any way.

Who can I contact for more information about magnesium sulphate therapy?

If you want more information about magnesium sulphate therapy, please talk to your doctor or midwife.

For further information

Antenatal Magnesium Sulphate Prior to Preterm Birth for Neuroprotection of the Fetus, Infant and Child National Clinical Practice Guidelines,

http://www.nhmrc.gov.au/publications/synopses/cp128syn.htm

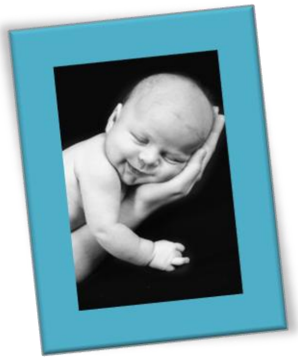

19/09/11

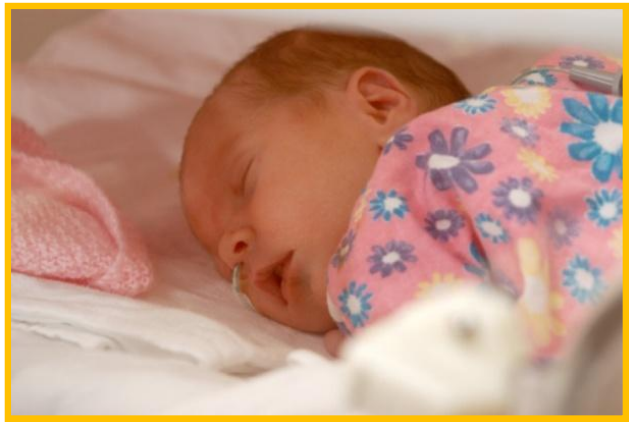

Antenatal magnesium sulphate therapy for improving the health of preterm babies

Information for Women
