# Supplementary material for: Working to improve survival and health for babies born very preterm: the WISH project protocol
Source: BMC Pregnancy Childbirth. 2013 Dec 19;13:239. doi: 10.1186/1471-2393-13-239 (PMC3879421; doi:10.1186/1471-2393-13-239)
Supplement: Additional file 4 — Magnesium sulphate implementation poster. These posters can be put up in perinatal care areas to assist with implementation of the clinical practice guidelines. [file 1471-2393-13-239-S4.pdf]

Antenatal magnesium sulphate ($MgSO_4$) prior to preterm birth for neuroprotection of the fetus, infant and child

WISH PROJECT

When to give $MgSO_4$?

☑ **Gestational age < 30 weeks**
☑ **Birth planned or definitely expected within 24 hours**

Give $MgSO_4$ regardless of :

- ☑ plurality
- ☑ parity
- ☑ reason at risk of preterm birth
- ☑ whether antenatal corticosteroids have been given or not
- ☑ anticipated mode of birth

What/When to administer?

$MgSO_4$ intravenously using a **dedicated** intravenous line:

- ☑ Commence $MgSO_4$ as close to four hours before birth as possible.
- ☑ Loading: 4g dose (slowly over 20-30 minutes).
- ☑ Maintenance: 1g/hour for up to 24 hours or until birth, whichever comes first.

When urgent delivery/birth needed:

☒ Do not delay delivery to administer $MgSO_4$

What if birth does not occur within 24 hours?

☑ Once 6 hours has transpired following the cessation of the 24 hour maintenance dose, a further loading and maintenance infusion may be considered.

How to monitor women?

- ☑ Monitoring is essential for both loading and maintenance doses.
- ☑ Monitor **pulse, blood pressure, respiratory rate** and **patellar reflexes**: (a) before loading infusion (b) 10 mins after starting infusion (c) after loading infusion is complete (d) every 4 hours during the maintenance infusion.
- ☑ Resuscitation and ventilator support should be available during and after administration of both magnesium sulphate and calcium gluconate.

When to stop $MgSO_4$ administration?

• Urine output <100mL in 4 hours • Absent patellar reflexes • Respiratory depression (< 12 breaths/min) • Hypotension (diastolic BP < 15 mm Hg below baseline).

→ **If Magnesium toxicity occurs:** Stop $MgSO_4$ infusion and administer antidote of calcium gluconate (10mL of 10% solution slowly intravenously over approx. 10 minutes).

Potential interactions between $MgSO_4$ and nifedipine may result in hypotension and neuromuscular blockade effects. If such interactions are evident, cease nifedipine and $MgSO_4$ infusion and seek medical review.

March 28th 2011
