# Supplementary material for: Working to improve survival and health for babies born very preterm: the WISH project protocol
Source: BMC Pregnancy Childbirth. 2013 Dec 19;13:239. doi: 10.1186/1471-2393-13-239 (PMC3879421; doi:10.1186/1471-2393-13-239)
Supplement: Additional file 5 — Case note reminder insert for magnesium sulphate. These case note inserts can be placed in the medical records of women at risk of very preterm birth to remind clinicians to consider their patient’s eligibility for antenatal magnesium sulphate. [file 1471-2393-13-239-S5.pdf]

WISH Project

Working to Improve Survival and Health for babies born very preterm

Is this woman eligible for magnesium sulphate for neuroprotection of her fetus?

- ☐ **< 30 weeks' gestation**
- ☐ **Birth planned or definitely expected within the next 24 hours**
- ☐ **Regardless of**
  - **Plurality**
  - **Parity**
  - **Reason for preterm birth**
  - **Anticipated mode of birth**
  - **Whether antenatal corticosteroids have been given or not**

Please see the poster overleaf *"Antenatal magnesium sulphate ($MgSO_4$) prior to preterm birth for neuroprotection of the fetus, infant and child"* for a quick reference on magnesium sulphate dosage, timing, monitoring and potential interactions.

28/03/2011

Antenatal magnesium sulphate ($MgSO_4$) prior to preterm birth for neuroprotection of the fetus, infant and child

WISH PROJECT

When to give $MgSO_4$?

☑ **Gestational age < 30 weeks**
☑ **Birth planned or definitely expected within 24 hours**

Give $MgSO_4$ regardless of :

☑ plurality
☑ parity
☑ reason at risk of preterm birth
☑ whether antenatal corticosteroids have been given or not
☑ anticipated mode of birth

What/When to administer?

$MgSO_4$ intravenously using a **dedicated** intravenous line:

☑ Commence $MgSO_4$ as close to four hours before birth as possible.
☑ Loading: 4g dose (slowly over 20-30 minutes).
☑ Maintenance: 1g/hour for up to 24 hours or until birth, whichever comes first.

When urgent delivery/birth needed:

☒ Do not delay delivery to administer $MgSO_4$

What if birth does not occur within 24 hours?

☑ Once 6 hours has transpired following the cessation of the 24 hour maintenance dose, a further loading and maintenance infusion may be considered.

How to monitor women?

☑ Monitoring is essential for both loading and maintenance doses.
☑ Monitor **pulse, blood pressure, respiratory rate** and **patellar reflexes**: (a) before loading infusion (b) 10 mins after starting infusion (c) after loading infusion is complete (d) every 4 hours during the maintenance infusion.
☑ Resuscitation and ventilator support should be available during and after administration of both magnesium sulphate and calcium gluconate.

When to stop $MgSO_4$ administration?

• Urine output <100mL in 4 hours • Absent patellar reflexes • Respiratory depression (< 12 breaths/min) • Hypotension (diastolic BP < 15 mm Hg below baseline).

→ **If Magnesium toxicity occurs:** Stop $MgSO_4$ infusion and administer antidote of calcium gluconate (10mL of 10% solution slowly intravenously over approx. 10 minutes).

Potential interactions between $MgSO_4$ and nifedipine may result in hypotension and neuromuscular blockade effects. If such interactions are evident, cease nifedipine and $MgSO_4$ infusion and seek medical review.

March 28th 2011
